# Changes in serum markers of patients with PCOS during consecutive clomiphene stimulation cycles: a retrospective study

**DOI:** 10.1186/s13048-019-0564-7

**Published:** 2019-10-04

**Authors:** Marlene Hager, Steffen Hörath, Peter Frigo, Marianne Koch, Rodrig Marculescu, Johannes Ott

**Affiliations:** 1 Department of Obstetrics and Gynecology, Clinical Division of Gynecologic Endocrinology and Reproductive Medicine, Medical University of Vienna, Spitalgasse 23, 1090 Vienna, Austria; 2 Department of Obstetrics and Gynecology, Clinical Division of General Gynecology and Gynecologic Oncology, Medical University of Vienna, Spitalgasse 23, 1090 Vienna, Austria; 3 Department of Laboratory Medicine, Medical University of Vienna, Spitalgasse 23, 1090 Vienna, Austria

**Keywords:** Polycystic ovary syndrome, Androgens, Ovarian stimulation, Clomiphene, Ovarian reserve

## Abstract

**Background:**

A retrospective case-control study was performed to evaluate whether PCOS-specific serum markers would change in women with polycystic ovary syndrome (PCOS) during the course of two consecutive cycles of clomiphene citrate (CC)-stimulation, which did not lead to a pregnancy.

**Methods:**

Anovulatory PCOS patients who underwent two consecutive CC-cycles (*n* = 41) and anovulatory PCOS controls who chose an observational approach for two months (*n* = 24) were included in the study. The main outcome measures were levels of follicle stimulating hormone (FSH), luteinizing hormone (LH), anti-Mullerian hormone (AMH), total testosterone, androstenedione, and sexual hormone binding globulin (SHBG).

**Results:**

In the control group, PCOS-specific serum parameters did not change during two months (*p* > 0.05). In the CC-group, there were decreases in LH (11.8 ± 4.9 mU/mL vs. 10.9 ± 4.0 mU/mL; *p* = 0.029), the LH:FSH ratio (2.1 ± 0.8 mU/mL vs. 1.8 ± 0.5 mU/mL; *p* = 0.007), and AMH (8.08 ± 4.27 ng/mL vs. 7.17 ± 3.37 ng/mL; *p* = 0.011), as well as an increase in SHBG (46.0 ± 20.2 nmol/L vs. 51.2 ± 21.0 nmol/L; *p* < 0.001). A higher age and lower baseline total testosterone and AMH levels were predictive of an AMH decline (*p* < 0.05).

**Conclusion:**

Two cycles of CC-stimulation that did not lead to a pregnancy were accompanied by mean LH, AMH, and LH:FSH ratio declines and an SHBG increase. The clinical significance seems of minor relevance.

## Background

Polycystic ovary syndrome (PCOS) is a commonly occurring endocrine disorder that affects 6–10% of women of reproductive age [1, 2]. In anovulatory PCOS women, clomiphene citrate (CC) is a first-line agent with which to induce ovulation [2, 3]. After stimulation with 50 mg CC, about 40–60% of PCOS women develop a follicle [4–6] and only about 30% of CC cycles result in a pregnancy [5]. In other words, women might not get pregnant due to failure of conception after ovulation induced by CC or due to CC resistance. The latter seems to be associated with a more severe PCOS phenotype, also suggested by higher anti Mullerian hormone (AMH) levels [7–9]. It has been hypothesized that these women would need higher FSH levels during ovarian stimulation in order to achieve response [10]. However, the majority of women will undergo more than one CC stimulation cycle. It has never been shown whether PCOS-specific serum markers would change after unsuccessful CC stimulation cycles. This could be of clinical importance, especially if CC stimulation leads to changes that are suggestive of worsening. This should also be considered against the background of a currently even more efficient alternative for ovulation induction in PCOS, namely letrozole [11–14].

Moreover, according to the recently posited “two triangles theory,” [15] follicle stimulating hormone (FSH) and AMH would be two of the key elements in PCOS pathogenesis. High AMH levels have been reported to predict CC resistance in PCOS [7, 8, 16], and, thus, might be suggestive of a more serious impairment of fertility as stated above. Moreover, CC treatment is accompanied by significant increases in circulating sex hormone binding globulin levels [17], which would then lower the levels of free testosterone. It seems plausible that influencing the system via CC stimulation could alter the endocrine milieu, either systemically, or locally, in the ovary.

Thus, it was the aim of this retrospective study to evaluate, in anovulatory women with PCOS, whether baseline levels of luteinizing hormone (LH), FSH, AMH, and androgens would change during the course of two cycles of CC stimulation that did not lead to a pregnancy, and to compare these patients to anovulatory women with PCOS who had chosen an observational approach.

## Results

There were no differences between the groups with regard to baseline patient characteristics (Table 1). In the CC stimulation group, 24 women (58.5%) had an ovulation after CC 50 mg. Thus, they underwent the subsequent stimulation with CC 50 mg again. The remaining 17 patients (41.5%) were resistant to CC 50 mg and received CC 100 mg in the next cycle. Of the latter, 10 women (58.8%) ovulated during the second stimulation cycle. In a first step, we analyzed the course of PCOS-specific serum parameters from baseline to the two-month follow-up in both groups (Table 2). There were no significant differences in the control group. In the CC stimulation group, significant decreases in LH, the LH:FSH ratio, and AMH were observed, whereas there was an increase in SHBG.

**Table 1:** Baseline patient characteristics of the CC and the control groups

Parameter	CC group (*n* = 41)	Control group (*n* = 24)	p
Age (years)	30.2 ± 5.9	30.0 ± 4.8	0.902
BMI (kg/m^2^)	25.6 ± 5.0	25.4 ± 5.4	0.893
Primary sterility	31 (75.6)	20 (83.3)	0.545
Nulliparity	36 (87.8)	22 (91.7)	1.000
TSH (μU/ml)	1.42 ± 0.96	1.79 ± 0.80	0.123
Estradiol (pg/mL)	60.71 ± 39.60	59.75 ± 29.07	0.613
Prolactin (ng/mL)	14.5 ± 8.9	13.5 ± 5.3	0.919
LH (mU/mL)	11.8 ± 4.9	13.0 ± 6.0	0.408
FSH (mU/mL)	5.9 ± 1.8	6.2 ± 1.5	0.518
LH:FSH ratio	2.1 ± 0.8	2.1 ± 0.8	0.885
Total testosterone (ng/mL)	0.43 ± 0.15	0.44 ± 0.15	0.804
Free testosterone (ng/mL)	0.30 ± 0.21	0.30 ± 0.19	0.936
Androstenedione (ng/mL)	2.77 ± 1.25	3.01 ± 1.13	0.446
SHBG (nmol/L)	46.0 ± 20.2	44.2 ± 27.3	0.767
AMH (ng/mL)	8.08 ± 4.27	8.07 ± 4.25	0.994
Dose of second CC stimulation cycle: 100 mg^a^	17 (41.5)	–	–

^a^versus 50 mg; all women in the CC group underwent an initial stimulation with 50 mg CC

Data are provided as mean ± standard deviation for numerical parameters or numbers (frequency) for categorical parameters; differences between groups were tested using unrelated t-tests for numeric parameters and Fisher’s exact tests for categorical parameters

**Table 2:** The course of PCOS-specific serum parameters in the CC and the control groups. Comparison of baseline to two-month follow-up values

Parameter	CC group (*n* = 41)				Control group (*n* = 24)			
	Baseline	After two months of CC stimulation	Delta	p	Baseline	After two months	Delta	p
LH (mU/mL)	11.8 ± 4.9	10.9 ± 4.0	−0.9 ± 2.8	0.029	13.0 ± 6.0	12.3 ± 5.5	− 0.68 ± 2.4	0.174
FSH (mU/mL)	5.9 ± 1.8	5.9 ± 1.4	0.1 ± 1.5	0.787	6.2 ± 1.5	5.5 ± 1.2	− 0.64 ± 1.7	0.072
LH:FSH ratio	2.1 ± 0.8	1.8 ± 0.5	− 0.2 ± 0.6	0.007	2.1 ± 0.8	2.1 ± 0.8	−0.05 ± 0.6	0.643
Total testosterone (ng/mL)	0.43 ± 0.15	0.43 ± 1.55	0.02 ± 0.11	0.651	0.44 ± 0.15	0.46 ± 0.12	0.02 ± 0.08	0.223
Free testosterone (ng/mL)	0.30 ± 0.21	0.27 ± 0.18	−0.03 ± 0.14	0.314	0.30 ± 0.19	0.26 ± 0.12	−0.03 ± 0.14	0.241
Androstenedione (ng/mL)	2.77 ± 1.25	3.23 ± 1.11	0.29 ± 0.76	0.216	3.01 ± 1.13	2.97 ± 0.93	−0.04 ± 0.80	0.801
SHBG (nmol/L)	46.0 ± 20.2	51.2 ± 21.0	5.2 ± 5.7	< 0.001	44.2 ± 27.3	43.0 ± 22.0	−1.2 ± 8.5	0.500
AMH (ng/mL)	8.08 ± 4.27	7.17 ± 3.37	−0.9 ± 2.1	0.011	8.07 ± 4.25	7.97 ± 4.18	−0.10 ± 1.1	0.654

Data are provided as mean ± standard deviation for numerical parameters or numbers (frequency) for categorical parameters; differences between groups were tested using paired t-tests

In a sub-analysis, we compared women who received CC 50 mg twice to those who were stimulated with CC 50 mg followed by CC 100 mg (Additional file 1: Table S1). The latter revealed a significant decrease in the LH:FSH ratio, whereas in women who had been stimulated with CC 50 mg twice a significant decline in AMH was found. Both subgroups revealed a significant SHBG increase.

We then analyzed possible predictive parameters for AMH and LH:FSH ratio dynamics in generalized linear models in the CC stimulation group (Table 3). A higher age and lower baseline total testosterone and AMH levels were significantly predictive of a decline in AMH (*p* < 0.05), whereas a higher baseline LH:FSH ratio and a lower baseline AMH level were associated with decreases in the LH:FSH ratio after two cycles of CC stimulation.

**Table 3:** Prediction of baseline- to two-month follow-up changes in AMH and the LH:FSH ratio. Results of the multivariate analysis using generalized linear models

	AMH dynamics		LH:FSH ratio dynamics	
	ß ± SD	p	ß ± SD	p
Age	−12.134 ± 6.884	0.078	0.006 ± 0.012	0.601
BMI	0.092 ± 0.067	0.169	0.021 ± 0.016	0.177
Baseline LH	−0.575 ± 0.466	0.217	0.137 ± 0.108	0.206
Baseline FSH	1.558 ± 1.000	0.119	−0.240 ± 0.232	0.301
Baseline LH:FSH ratio	3.967 ± 2.521	0.116	−1.298 ± 0.586	0.027
Baseline SHBG	0.038 ± 0.021	0.067	0.009 ± 0.005	0.069
Baseline total testosterone	8.664 ± 3.902	< 0.001	0.014 ± 0.565	0.980
Baseline AMH	−0.435 ± 0.071	< 0.001	0.038 ± 0.017	0.023
Constant	−12.134 ± 6.884	0.078	0.804 ± 1.600	0.615

## Discussion

The general patient characteristics quite closely represented the average PCOS population [9, 18]. Moreover, there were no differences between the CC group and the control group. Thus, the observed groups were comparable despite the lack of matching. This is a minor study limitation that is attributable to the fact that only a minority of women chose an observational approach or wanted to postpone treatment initiation.

The main findings of this study were the decreases in AMH and the LH:FSH ratio after two CC cycles that did not lead to a pregnancy. Both markers are thought to somehow represent the severity of PCOS [18]. It is difficult to state whether these decreases could be beneficial or unfavorable. On the one hand, a decline could be considered an improvement in PCOS severity, probably also in terms of ovarian response to FSH secretion after CC stimulation, since higher AMH levels might serve as predictors for CC resistance [7–9, 16]. On the other hand, the reduction of circulating AMH might also represent damage to the ovarian reserve. The latter has at least been discussed with regard to ovarian drilling [19]. Comparable studies revealed either relative AMH declines during recombinant FSH treatment [20] or significant absolute AMH declines in PCOS women with a baseline AMH ≥5 ng/ml during mono-ovulation induction with either antiestrogens or recombinant FSH [21]. Notably, the observed AMH decreases “might have derived from the recruitment of small AMH-producing follicles to growing follicles, stopping AMH secretion” [21]. Accordingly, reduced AMH expression following FSH treatment has already been demonstrated in vitro [22]. This might also be reflected by the fact that, in our data set, the AMH decline was only significant in women who had ovulated after the initial CC stimulation with 50 mg and not in those who were resistant to it (Additional file 1: Table S1).

The observed AMH and LH:FSH ratio decreases reflect a close association, which has already been reported [23, 24]. Experimental data have suggested a possible stimulating effect of AMH on gonadotropin-releasing hormone neuron activity and secretion [25]. Thus, one might assume that the decline in LH was secondary to that in AMH. However, the decline in the LH:FSH ratio was only evident in women refractory to CC 50 mg, which we find hard to interpret. Since the dynamics in the LH:FSH ratio were primarily due to the LH decline (Table 1), the latter might be seen as an effect of luteal support in our patient population. However, the half-lives of dydrogesterone and its metabolite, 20-dihydrodydrogesterone, are only 5–7 h and 14–17 h, respectively [https://pubchem.ncbi.nlm.nih.gov/compound/dydrogesterone#section=Metabolism-Metabolites]. All follow-up serum blood samples were retrieved on the 2nd to 5th cycle day after a withdrawal bleeding. Thus, we consider a residual suppression effect on LH unlikely. We can rule out that the observed changes might be due to ovulation induction with recombinant human chorionic gonadotropin (rHCG), since a previous study showed no impact of rHCG administration in PCOS women on AMH up to 48 h thereafter [23].

In the present dataset, the AMH declines were associated with lower total testosterone levels, as well as higher age and baseline AMH levels (Table 3). The latter would be in line with the above-cited study about AMH levels during mono-ovulation induction [21]. It is worth mentioning that older women also seemed more prone to AMH declines. A gradual decline of about 5.6% per year with advancing age has been reported [26]. In our dataset, there was a decline in mean AMH of about 11% [8.08 ng/mL versus 7.17 ng/mL], which we consider more than merely marginal. However, its clinical relevance seems difficult to assess.

The decline in the LH:FSH ratio could have been predicted by the higher baseline LH:FSH ratio and a lower baseline AMH level, with an only modest effect of the latter (0.038 ± 0.017; Table 3). CC clearance from the circulation can take up to two months [27] and, thus, a residual CC effect on FSH might have been responsible. However, FSH remained stable after CC stimulation (Table 2) which seems to disprove this hypothesis. The SHBG-increasing effect of CC is well known [17], and was also observed in our dataset. This is likely due to the above-mentioned residual action of CC. In addition to the already mentioned study limitations, a few additional issues should be considered, including the small sample size and the retrospective approach.

## Conclusions

To the best of our knowledge, this is the first study on the course of PCOS-specific serum parameters after more than one CC stimulation cycle. Two cycles resulted in mean LH, AMH, and LH:FSH ratio declines and an increase in mean SHBG. The observed effects of CC stimulation on the hormonal profile seem of minor clinical relevance.

## Materials and methods

The Clinical Division of Gynecologic Endocrinology and Reproductive Medicine of the Medical University of Vienna, Austria, serves as a referral center for gynecologic endocrinology and reproductive medicine. The diagnosis of PCOS was confirmed according to the revised Rotterdam criteria of 2004 [28]. All women demonstrated ≥12 follicles of 2-9 mm diameter on at least one ovary on transvaginal ultrasound, as well as 17-hydroxy progesterone levels < 2 ng/mL, which excluded non-classic adrenogenital syndrome. From June 2016 to February 2018, 124 anovulatory PCOS women were stimulated with CC at the department. For this retrospective study, women who were included in the CC (case) group had undergone at least two consecutive cycles of CC, starting with an initial dose of 50 mg (*n* = 90). The following exclusion criteria were applied: (i) use of co-medications, i.e.*,* metformin, myo-inositol, cortisone/prednisolone, or any study-specific medications (*n* = 28); (ii) any ovarian stimulation within the last three months (*n* = 3); and (iii) women who had become pregnant after the second CC cycle or had chosen not to undergo any treatment in the subsequent cycle, since no third pre-stimulation hormonal blood-testing was available in these cases (*n* = 18). Thus, 41 women were included in the CC group.

For the control group, we identified 24 anovulatory PCOS women who did not undergo ovarian stimulation or PCOS-specific treatment for two months after initial hormonal testing, but delayed treatment by request. Selected women were diagnosed within the period mentioned above and underwent a second pre-stimulation hormonal testing after two months. None of them had undergone any previous ovarian stimulation nor were any of them included in the CC group in case of a later CC stimulation. None of the patients had undergone previous ovarian drilling.

For the CC group, menstrual bleeding was induced through oral administration of 20 mg dydrogesterone for ten days. Subsequently, CC stimulation was done as published previously [29] and began with an initial dose of 50 mg/day from the 5th to the 9th day of the menstrual cycle. In case of resistance to 50 mg CC, defined as the inability to develop a leading follicle, 100 mg/day from the 5th to the 9th day were administered in the second cycle. Beginning with the 10th day, patients underwent daily sonographic monitoring of follicular growth. If the leading follicle reached a mean diameter of 19 mm, ovulation was induced by intramuscular application of HCG. Then, all women received dydrogesterone 20 mg, administered for 10 days beginning on the day after ovulation induction.

Pre-stimulation hormonal testing was performed on the 2nd to 5th day of each stimulation cycle to obtain the most recent predictive parameters. Although this is not necessary according to established guidelines [2, 12], this has been part of the clinical routine at this department.

In the control group, bleeding was also induced by administration of 20 mg oral dydrogesterone, as described above. Thus, all hormonal parameters were retrieved on the 2nd to 5th cycle day.

We included the following parameters: patients’ age at the first hormonal testing used in this study; body mass index (BMI); type of sterility (primary/secondary); parity; initial baseline serum levels of thyroid stimulating hormone (TSH), prolactin, and estradiol; and serum levels of FSH, LH, AMH, total testosterone, androstenedione, and sexual hormone binding globulin (SHBG), measured initially and after two CC cycles (CC group) or two months of observation (control group). All hormone measurements were performed in ISO 9001 certified and ISO 15189 accredited clinical diagnostic laboratories at the Department of Laboratory Medicine, Medical University of Vienna. IVD CE certified electrochemiluminescence immunoassays (ECLIA) on Cobas e 602 immunology analyzers (Roche Diagnostics, Vienna, Austria) were used for all parameters, except androstenedione. Intra- and inter-assay coefficients of variation (CV) were ≤ 11.1 and 11.9% for TSH (normal range: 0.44–3.77μU/mL), 1.7 and 2.0% for prolactin (normal range: 4.8–23.3 ng/mL), 6.7 and 10.6% for estradiol (baseline normal range: 12.5-166 pg/mL), 2.8 and 4.5% for FSH (baseline normal range: 3.5–12.5 mU/mL), 1.2 and 2.2% for LH (baseline normal range: 2.4–12.6 mU/mL), 1.7 and 3.5% for AMH (normal range: 0–12.6 ng/mL), 14.8 and 18.1% for total testosterone (normal range: 0.08–0.48 ng/mL), and 1.7 and 4.0% for SHBG (normal range: 26.1–110.0 nmol/L), respectively. Androstenedione determinations were performed by an IVD CE certified chemiluminescence immunoassay (CLIA) on an Immulite 2000 XPi immunology analyzer (Siemens Healthcare Diagnostics Products, Vienna, Austria) with intra-assay and total CV ≤ 15.1 and 17.8% (normal range: 0.3–3.7 ng/mL), respectively. Free testosterone was calculated according to the algorithm published by Vermeulen et al. [30].

Variables are described by numbers (frequencies) for categorical parameters, mean ± standard deviation for normally distributed numerical parameters and median (interquartile range) for non-normally distributed numerical parameters. Statistical analysis was performed with SPSS 24.0 for Windows (SPSS Inc., 1989–2018) using unrelated t-tests and paired t-tests (for normally distributed numeric parameters), Wilcoxon signed rank tests (for non-normally distributed numeric parameters) as well as the Chi-square test or the Fisher’s exact test (for categorical parameters). Possible influencing factors on hormone dynamics were tested using generalized linear models. For these, regression coefficients (ß) and their standard deviations (SD) are provided. Differences were considered statistically significant if *p* < 0.05.

## Supplementary information

**Additional file 1: Table S1.** The course of PCOS-specific serum parameters in the group of women who received CC 50 mg twice and the group of women who received CC 50 mg followed by 100 mg.

## Data Availability

The datasets generated and/or analyzed during the current study are not publicly available, since the dataset will be used for other retrospective analyses. The data are available from the corresponding author upon reasonable request.
