# Supplementary material for: Changes in serum markers of patients with PCOS during consecutive clomiphene stimulation cycles: a retrospective study
Source: J Ovarian Res. 2019 Oct 4;12:91. doi: 10.1186/s13048-019-0564-7 (PMC6777034; doi:10.1186/s13048-019-0564-7)
Supplement: Supplementary file 1 — Additional file 1: Table S1. The course of PCOS-specific serum parameters in the group of women who received CC 50 mg twice and the group of women who received CC 50 mg followed by 100 mg. [file 13048_2019_564_MOESM1_ESM.docx]

**Additional file 1: Table S1**. *The course of PCOS-specific serum parameters in the group of women who received CC 50mg twice and the group of women who received CC 50mg followed by 100mg.*

| Parameter | CC 50mg followed by 50mg  (n= 24) | | | CC 50mg followed by 100mg  (n= 17) | | |
| --- | --- | --- | --- | --- | --- | --- |
|  | Baseline | After two months of CC stimulation | p | Baseline | After two months | p |
| LH (mU/mL) | 9.7 (7.4;14.8) | 8.9 (7.5;13.4) | 0.385 | 11.3 (8.9;16.8) | 10.4 (8.9;14.7) | 0.052 |
| FSH (mU/mL) | 6.0 (4.8;7.4) | 6.1 (5.1;7.3) | 0.973 | 5.2 (4.4;5.9) | 5.5 (5.1;6.1) | 0.524 |
| LH:FSH ratio | 2.0 (1.3;2.2) | 1.9 (1.2;2.1) | 0.104 | 2.1 (1.6;3.1) | 1.8 (1.6;2.6) | 0.036 |
| Total testosterone (ng/mL) | 0.35 (0.28;0.46) | 0.37 (0.29;0.44) | 1.000 | 0.48 (0.40;0.59) | 0.50 (0.38;0.59) | 0.291 |
| Free testosterone (ng/mL) | 0.16 (01.4;0.28) | 0.17 (0.15;0.29) | 0.362 | 0.34 (0.22;0.41) | 0.28 (0.19;0.34) | 0.125 |
| Androstenedione (ng/mL) | 2.41 (1.56;3.27) | 2.96 (2.14;4.22) | 0.252 | 3.41 (2.20;3.95) | 3.75 (2.85;4.51) | 0.695 |
| SHBG (nmol/L) | 57.4 (33.5;64.8) | 57.8 (37.6;69.7) | 0.002 | 31.6 (22.8;55.4) | 40.3 (30.7;57.7) | <0.001 |
| AMH (ng/mL) | 6.35 (5.27;7.34) | 5.78 (4.61;7.04) | 0.006 | 8.24 (5.90; 14.11) | 8.50 (5.82;10.92) | 0.132 |

Data are provided as median (interquartile ranges) for numerical parameters or numbers (frequency) for categorical parameters; differences between groups were tested using Wilcoxon signed rank tests.
